# Igg-Dependent Hydrolysis of Myelin Basic Protein of Patients with Different Courses of Schizophrenia

**DOI:** 10.1155/2020/8986521

**Published:** 2020-08-10

**Authors:** Daria A. Parshukova, Liudmila P. Smirnova, Elena G. Kornetova, Arkadiy V. Semke, Valentina N. Buneva, Svetlana A. Ivanova

**Affiliations:** ^1^Laboratory of Molecular Genetics and Biochemistry, Mental Health Research Institute, Tomsk National Research Medical Center of the Russian Academy of Sciences, Tomsk 634014, Russia; ^2^Laboratory of Repair Enzymes, Institute of Chemical Biology and Fundamental Medicine, Siberian Branch of the Russian Academy of Sciences, Novosibirsk 630090, Russia

## Abstract

The level hydrolysis of myelin basic protein (MBP) by IgG in patients with schizophrenia was studied depending on the clinical features and course of the disease. The patients were grouped according to type of schizophrenia and type of disease course. We found that IgGs isolated and purified from sera of schizophrenia patients' blood hydrolyses human MBP, and the level of this hydrolysis significantly exceeds that of healthy individuals. Detection of protease activity corresponding only to intact IgGs in polyacrylamide gel fragments, together with data of gel filtration of antibodies under conditions of “acid shock” (concordance of optical density profile of IgG with profile of MBP-hydrolyzing activity) and with the absence of any other proteins and bands in gradient SDS-PAGE and in PVDF membrane provides direct evidence that the IgGs from the schizophrenia patients have MBP-hydrolyzing activity. The antibodies-specific proteolytic activity of patients with acute schizophrenia (1.026 [0.205; 3.372] mg MBP/mg IgG/h) significantly exceeds the activity of IgG in patients in remission (0.656 [0.279; 0.873] mg MBP/mg IgG/h) and in healthy individuals (0.000 [0.00; 0.367] mg MBP/mg IgG/h). When comparing the specific activity in patients with different types of disease course, we have found that patients with a continuous course of paranoid schizophrenia (1.810 [0.746; 4.101 mg MBP/mg IgG/h]) had maximal activity values. It can be assumed that the increase in the activity of MBP-hydrolyzing antibodies is due to the activation of humoral immunity in acute schizophrenia.

## 1. Introduction

The interconnection between the nervous and immune systems has been recognised in recent years but many questions remain regarding their interactions in schizophrenia. The idea of an autoimmune component in the pathogenesis of schizophrenia was first proposed by Hermann Lehmann-Facius in 1937, and it was further developed by others [1–4].

In schizophrenia, the inhibition of regulatory T-lymphocytes was revealed, this leading to the activation of humoral immunity and resulting in the formation of antibodies to various components of the nervous tissue [5, 6]. It was shown in a BALB/c mouse model that autoantibodies (Abs) binding neuroantigens in the early stages of ontogeny promote the occurrence of various structural anomalies in the nervous system, and this subsequently leads to inhibitory effect on physical development, training processes, and memory [7, 8].

Postmortem studies in patients with schizophrenia have revealed deficits in myelination, abnormalities in myelin gene expression, and altered numbers of oligodendrocytes in the brain [9]. Studies in adult postmortem samples using electron microscopy and histochemistry suggest structural changes in oligodendroglia cells that produce the myelin sheath [10, 11], an atrophy of axons, abnormalities of the myelin sheath surrounding [12, 13], a reduction in myelin basic protein (MBP) in the anterior frontal cortex in cases of schizophrenia [14], and a decrease in MBP expression in the gray matter of the frontal cortex of patients with schizophrenia [15]. MBP is one of the main protein components of the central nervous system myelin, and it reflects the myelin-forming activity of oligodendroglia through a positive correlation with the number of normal myelin fibres [16, 17].

Antibodies to MBP in the serum of patients with schizophrenia were found long ago [18]. An increase in the level of antibodies to neuroantigens, including MBP-antibodies, in schizophrenia correlates with the severity of clinical symptoms [19]. Increased antibody levels are often interpreted as a breakdown of immune tolerance, causing an autoimmune response [20]. But at the same time, this may reflect a hyperactivity of the immune system as part of the etiology of schizophrenia, in accordance with literature data about an increase in proinflammatory molecules with increased immune response in those undergoing both acute psychosis and chronic schizophrenia [21–23].

The existence of catalytic antibodies (so-called abzymes) has been known for over 30 years. The ability of immunoglobulins to catalyze many chemical reactions, e.g., hydrolysis of DNA, RNA, polysaccharides, and proteins has been described. Abzymes capable of hydrolyzing MBP were found and studied in detail in multiple sclerosis, systemic lupus erythematosus [24, 25], and in the serum of autistic children [26].

Recently, we reported an ELISA study showing that titers of autoantibodies against MBP in patients with schizophrenia are ~1.8 fold higher than in healthy individuals, but 5.0-fold lower than in patients with multiple sclerosis. More importantly, we also reported that such antibodies had abzyme (catalytic) activity, meaning that they were also capable of hydrolyzing MBP and its peptides [27]. In this report, we present data on the catalytic properties of the MBP-hydrolyzing abzymes in sera from patients having a more diverse diagnostic profile in comparison with the previous study.

In our previous study, it was shown that the greatest MBP-hydrolyzing activity is associated with negative symptoms and a long course of the disease, which allows us to suggest a connection between the severity of clinical symptoms and damage of myelin, causing an increase in the level of proteolytic activity of antibodies. Therefore, the purpose of this study was to investigate the characteristics of MBP proteolysis by serum polyclonal antibody (abzymes) of schizophrenia patients depending on the clinical features of the disease.

## 2. Materials and Methods

### 2.1. Characteristics of the Studied Subjects

In this work, 79 patients (61% males, 39% females) with schizophrenia were recruited to study the proteolytic activity of their antibodies. The age of patients varied from 21 to 61 years with a median 36.00 [31.00; 45.00] years. Inclusion criteria were the following: paranoid or simple schizophrenia according to the International Statistical Classification of Diseases and Related Health Problems, 10th Revision (ICD-10: F20.0 and F20.6), and the Structured Clinical Interview for DSM-IV Axis I Disorders (SCID). Exclusion criteria were the following: the presence of acute or chronic infectious, inflammatory, autoimmune or neurological diseases, other organic mental disorders, and mental retardation. The schizophrenia diagnosis was confirmed and verified in accordance with the international standard criterion, the psychometric PANSS scale.

The sample was formed from inpatients hospitalized due to clinical symptoms (hereinafter “acute schizophrenia”) and outpatients in remission. Of these, 48 patients exhibited signs, according to ICD-10 criteria, for paranoid schizophrenia (continuous F20.00, episodic with progressive deficit F20.01, episodic with stable deficit F20.02) and simple schizophrenia F20.6. These patients received treatment at the Mental Health Research Institute TNMRC, Department of Endogenous Disorders (Tomsk, Russia). A total of 31 patients were assigned to a group with clinical remission (F20.05). Patients with clinical remission were invited to the study by their doctors of the Department of Endogenous Disorders. Data on the participants are presented in Tables 1 and 2.

The level of education of patients was as follows: higher—25 people (32%), incomplete higher—10 (12%), secondary special—27 (34%), secondary—17 (22%). Among the patients in the study sample, the majority were not married—55 people (70%), 16 (20%) married, 3 (4%) divorced, 2 (2%) in a civil marriage, and 3 people were widows (4%). Most patients received second-generation antipsychotics in maintenance dosages before admission to the hospital (olanzapine, 20 patients (25.32%), quetiapine, 24 (30.38%), risperidone, 30 (37.98%), or clozapine, 5 (6.3%)). They were often nonadherent, and thus were hospitalized due to exacerbation of symptoms of schizophrenia. Blood sampling was performed at hospitalization before the administration of antipsychotic therapy.

All individuals included in the study gave written informed consent. Ethical approval was granted (protocol N 78/1.2015) by the Local Bioethics Committee of the Mental Health Research Institute in accordance with Helsinki ethics committee guidelines. None of the participants were compromised in their capacity/ability to consent; thus, consent from the next-of-kin was not necessary, and it was not recommended by the local ethics committee.

The control group consisted of 24 subjects (38% males, 62% females). In this group, age varied from 23 to 53, with a median 39.00 [29.00; 46.00] years. These control subjects were mentally and somatically healthy individuals. The excluding criteria for the controls were the presence of acute and chronic infectious, inflammatory, autoimmune, or neurological diseases, and organic brain disorders.

### 2.2. Object of Study

Blood samples were obtained after an overnight fast from a vein into tubes with a clot activator (CAT, BD Vacutainer). To isolate the serum, the blood samples were centrifuged for 30 min at 2000 × g at 4°C. The sera were stored at −80°C until analysis.

### 2.3. Purification of IgGs

Affinity chromatography on a chromatographic matrix with immobilized protein G from group G streptococci is a widely used and effective method for the isolation of immunoglobulins. Protein G has a high affinity for the Fc regions of immunoglobulins G. This property allows selective elution of components of the immune complexes (proteins, polysaccharides, nucleic acids) under conditions with increased ionic strength or in the presence of nonionic detergents without destroying the Ig complexes with protein G [28]. Antibodies from the blood sera of schizophrenia patients and healthy controls were purified and analyzed by earlier developed procedures for purification of electrophoretically and immunologically homogenous IgG preparations from human blood serum [29, 30]. The procedure included affinity chromatography of serum proteins on Protein G-Sepharose, followed by high-performance gel filtration on a Superdex-200 HR 10/30 column. Quantitative elution of IgG was carried out using an acid buffer with a pH of 2.6, after which the resulting sample was immediately neutralized.

### 2.4. SDS-PAGE Analysis of Proteins

Electrophoretic separation of proteins according to the Laemmli method [31] was used to analyze the homogeneity of antibodies as well as to analyze the products of hydrolysis of MBP by Abs. The concentrating gel contained 4% acrylamide (AA: Bis-AA ratio = 30 : 1), 125 mM Tris-HCl, pH 6.8, and 0.5% SDS. The separating gel contained 5-20% AA, 375 mM Tris-HCl, pH 8.8, and 0.4% SDS. The protein preparations were incubated in buffer containing 50 mM Tris-HCl, pH 6.8, 2% SDS, 10% glycerol, 0.025%, and bromophenol blue at 100°C for 1 min and then applied to the gel. The electrophoresis was performed at the current 15-20 A. Homogeneity of the Abs was tested in 4–15% gradient gels (0.1% SDS). The polypeptides were visualized by silver or Coomassie R250 staining and by Western blotting on a nitrocellulose membrane [32]. The gels were imaged by scanning and quantified using the Image Quant 5.2 program.

### 2.5. FPLC Gel Filtration under “Acid Shock” Conditions

Acidic pH of the medium allows dissociation and subsequent separation of all components of immunocomplexes consisting of immunoglobulins and their associated antigens. Electrophoretically homogeneous preparations of IgG were preincubated in glycine buffer (pH 2.6) followed by gel filtration in the same buffer (acid shock). The IgG was separated by high-performance gel filtration on a Superdex 200 HR column equilibrated with 50 mM glycine-HCl (pH 2.6) containing 0.1 M NaCl (buffer B) using Akta Pure chromatography (GE). The IgG samples (150 *μ*l, 20 mg/ml) were preincubated with 50 *μ*l of buffer B; then, the high-performance gel filtration was performed at 22°C. The resulting fractions were immediately neutralized with 1 M Tris-HCl buffer (pH 8.8). The eluate was sequentially collected in individual tubes (Eppendorfs) at a volume of 1 ml. Each tube with 1 ml of antibody preparation is a separate fraction of the IgG preparation. The concentration of IgG preparations was determined at a wavelength of *λ* = 280 nm, in quartz cuvettes with 1 cm optical path length against a buffer in which IgG was dissolved on an Eppendorf BioPhotometer (Germany) single-beam spectrophotometer. After one week of storage at 4°C for refolding after the acid shock, the IgG was used in activity assays as described below.

This work was performed at the laboratory of Repair Enzymes of the Institute of Chemical Biology and Fundamental Medicine at the Siberian Branch of the Russian Academy of Sciences in Novosibirsk under the guidance of V. Buneva.

### 2.6. In Situ Proteolytic Activity Assay

This approach gives an indication of the enzymatic activity of a specific fragment of a gel or a specific protein band. One of the options for this approach is to determine the activity of enzymes in the eluates of various fragments of the gel. After standard SDS-PAGE of IgGs, to restore the MBP-hydrolyzing activity of IgGs, SDS was removed by incubation of the gel for 1 h at 300 C with 4 M urea and washed 10 times (7-10 min) with H_2_O. Then 2-4 mm cross-sections of longitudinal slices of the gel were cut up and incubated with 50 *μ*l of 50 mM Tris-HCl, pH 7.5, containing 50 mM NaCl for 6-7 days at 40°C to allow protein refolding and eluting from the gel. The solutions were removed from the gels by centrifugation and used for assay of MBP hydrolysis as described below. Parallel control longitudinal lanes were used to detect the position of intact IgG as well as its light and heavy chains after Ab reduction on the gel by silver staining.

This work was performed at the laboratory of Repair Enzymes of the Institute of Chemical Biology and Fundamental Medicine at the Siberian Branch of the Russian Academy of Sciences in Novosibirsk under the guidance of V. Buneva.

### 2.7. Proteolytic Activity of Immunoglobulin G Fractions Purified from Serum

The purified IgG was tested for MBP-hydrolyzing activity with MBP isolated from human brain tissue. The MBP was obtained from the Department of Biotechnology, Research Center of Molecular Diagnostics and Therapy (Moscow). The reaction mixture (10-40 *μ*l) for analysis of MBP-hydrolyzing activity of IgG contained IgG in concentration 0.2 mg/ml, 20 mM Tris-HCl (pH 7.5), and 0.2-0.7 mg/ml MBP. The products of MBP cleavage were analyzed in 4-15% or 12% SDS-PAGE as described earlier in Section 2.5. All quantitative measurements (initial rates) were taken under pseudo-first-order conditions of the reaction within the linear region of Abs concentrations, of the time courses (1-24 h), and the formation of products (15-40% of MBP hydrolysis). The catalytic activity of the IgG in the cleavage of MBP was estimated from the decrease in the intensity of the Coomassie-stained MBP band after electrophoresis. Differences in the hydrolysis levels of MBP incubated in the absence and in the presence of IgG were used to correct the values. Quantitative evaluation of proteins was estimated using the Image Quant 5.2 program. The activity of the Abs is expressed in units of specific enzyme activity as the quantity of substrate cleavage by 1 mg Abs per/h.

### 2.8. Statistical Analysis

Statistical analyses were performed with Statistica 10.0 software for Windows. The data were checked for normal distribution using the Shapiro–Wilk *W* test. Most of the sample sets did not meet the normal Gaussian distribution. For this reason, the differences between IgG samples of different groups were estimated using the Mann–Whitney test and the Kruskal–Wallis Test; a difference was considered statistically significant at *p* < 0.05. The median (*M*) and interquartile ranges (IQR) were estimated.

## 3. Results and Discussion

In this study, we analyzed Abs (IgGs), having MBP-hydrolyzing activity, purified from sera of patients with schizophrenia and healthy controls. The important tasks of our work with catalytic antibodies were to show that the studied activity belonged to the antibodies, but not to simultaneous-obtained competing proteases. It was proven that checking the three most stringent, indicative, and reliable criteria is sufficient to unambiguously conclude that the studied catalytic activity belongs to isolated antibodies. The efficiency of substrate hydrolysis by antibodies belonging to different subclasses may be different. Given this, we decided to study the content of IgG subclasses with proteolytic activity in the groups of schizophrenia patients.

### 3.1. Application of Strict Criteria for IgGs with Proteolytic Activity

Several strict criteria were tested to show that detected catalytic activity belonged to the antibodies: purification of Abs on sorbent with affinity to IgG, electrophoretic homogeneity of antibodies in SDS-PAGE, gel filtration chromatography of Abs under conditions of dissociation of immune complexes (pH shock analysis), and proteolytic activity in situ. It turned out that checking of these most stringent, indicative, and reliable criteria is sufficient to unambiguously conclude that the studied catalytic activity belongs to the Abs. These criteria for checking the presence of catalytic activity in antibodies are generally accepted and are used by various independent research teams [26, 28, 33–36].

Based on the specific binding of isolated IgG to Protein G-Sepharose sorbent, the catalytic activity of IgG is directly shown.

The isolated IgG preparations were electrophoretically and immunologically homogeneous in accordance with silver staining and immunoblotting after separation in gradient SDS-PAGE and transferred to the PVDF membrane.

**Figure 1:**
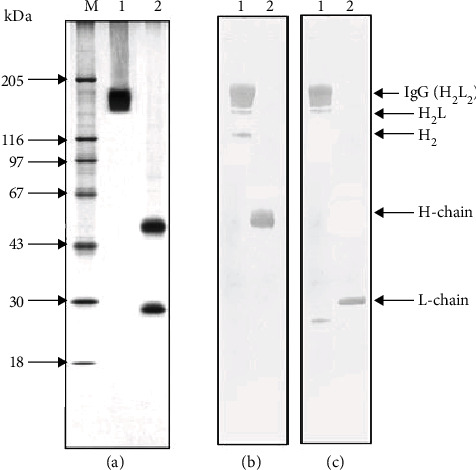
Analysis of homogeneity of IgG preparations using SDS-PAGE in 5–18% gradient gel and Western blot. (а) Silver staining: IgG preparation before (lane 1) and after (2) incubation with 10 mM DTT (membrane stained with silver). (b) Western blot analysis after incubation with horseradish peroxidase conjugates with rabbit anti-H IgG: 1 and 2, before and after incubation with 10 mM DTT, respectively. (c) Western blot analysis after incubation with a horseradish peroxidase conjugates with rabbit anti-L IgG: 1 and, 2 before and after incubation with 10 mM DTT, respectively. М—protein molecular mass markers.

The homogeneity of the 150 kDa IgG was confirmed by 4-18% SDS-PAGE, which showed a single protein band corresponding to the molecular mass of IgG (Figure 1(a)). The electrophoretic mobility of usually low molecular mass canonical proteases (24-25 kDa) cannot coincide with that of intact IgG (150 kDa). A major band 150 kDa corresponding to the whole IgG molecule consisting of two light and two heavy chains (H2L2) is visible (lane 1), also after incubation with 10 mM DTT (membrane stained with silver) bands corresponding to the light chain (25 kDa) and heavy chain of IgG (50 kDa) (lane 2).

Western blot analysis (Figure 1(b)) reveals additional bands—oligomeric forms of IgG of H2 composition. Partial decomposition of the Abs into their subunits L and H2 subunits in the presence of SDS is due to disulfide exchange [37]. Since the IgG molecule has two disulfide bonds between heavy chains and only one covalent bond is formed between the L and H chains, the probability of separation of the light chain from the IgG molecule in the presence of a denaturing agent is theoretically higher than the formation of HL dimers. Only in the IgG4 subclass, whose share is 3–4% of the total IgG pool, one S-S bond is formed between the heavy chains, and it is characterized by high lability [38].

**Figure 2:**
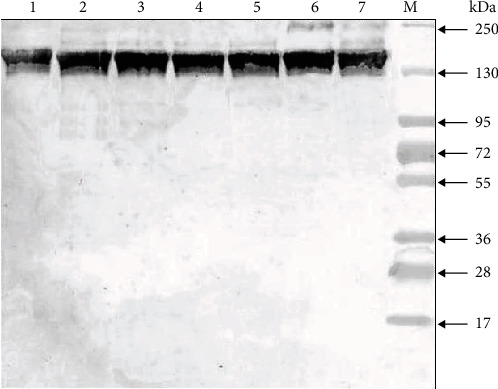
Analysis of homogeneity of IgG preparations after SDS-PAGE in a 5–18% gradient gel and silver staining. Lines 1-7—IgG of different schizophrenia patients; М—protein molecular mass markers.

Thus, in the SDS-PAGE analysis of the total IgG preparation, all oligomeric forms can be observed as minor components: H2L, HL, and H2, as well as the free L chain. The mobility of the light chain decreases after the restoration of intra-chain S-S bonds, which is explained by the complete unfolding of the polypeptide chain and a decrease in its compactness. We also present an analysis of the electrophoretic homogeneity of IgG in several schizophrenic patients with silver stain (Figure 2).

**Figure 3:**
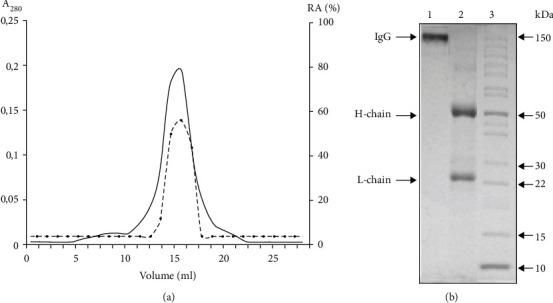
(a**)** Gel filtration of IgG on a Superdex 200 column in acidic buffer (pH 2.6) after preincubation of the Abs in the same buffer: (—). absorbance at 280 nm (A280) that reflects the content of IgG; (■). Relative activity (RA. %) of IgGs in the hydrolysis MBP. The complete hydrolysis of MBP for 5 h was taken for 100%. (b) Electrophoretic analysis in gradient PAGE (4.5–15%) of the fraction corresponding to the central part of the peak (Figure 2(a)) before (line 1) and after (line 2) incubation with a reducing agent (10 mM DTT). Line 3—protein molecular mass markers.

One of the most important criteria for attributing the activity to Abs is gel filtration of the Abs under acidic conditions, where noncovalent complexes are dissociated. Electrophoretically homogeneous preparations of IgG were preincubated in glycine buffer (pH 2.6) followed by gel filtration in the same buffer (acid shock). After the gel filtration, we obtained 25 fractions of one IgG preparation with different antibody concentrations. To assess the level of antibody activity in MBP hydrolysis, we took the fraction corresponding to the peak of the chromatogram. Standard reaction conditions used with high-performance gel filtration of the IgGs under acid-shock conditions (pH 2.6) demonstrated the concordance of the optical density profile (*λ* = 280 nm) with the profiles of the MBP-hydrolyzing activity, which is another indication of the studied activity being due to the IgGs (Figure 3).

**Figure 4:**
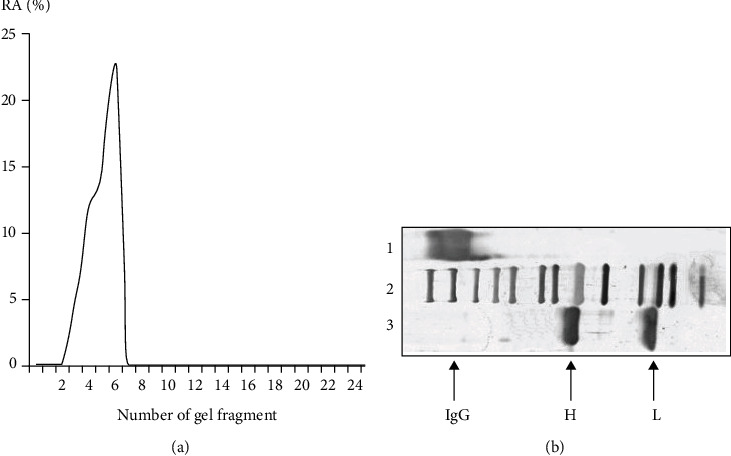
(a) Graph of MBP-hydrolyzing activity of extracts from various gel fragments after SDS-PAGE of an IgG preparation. (b) Electrophoretic analysis of IgG protein. Assay of MBP-hydrolyzing activity of purified IgG after SDS-PAGE in 4–15% gradient gel; the gel was incubated under special conditions for renaturation of the Abs. The relative proteolytic activity (RA. %) was revealed using extracts of many 2–3 mm fragments of one longitudinal slice of the gel. The RA of IgG corresponding to complete hydrolysis of 0.5 mg/mL MBP after incubation for 24 h with 10 *μ*l of the extract was taken for 100%. The average error in the initial rate determination from three experiments did not exceed 10–15%. The second set of control longitudinal slices of the same gels corresponding to IgGs before (lane 1) and after (lane 3) incubation with DTT was stained with silver. Lane 2 shows the position of molecular mass markers. The IgG—band is in the region of 150 kDa, which corresponds to the native IgG; the H—band is in the region of 50 kDa, which corresponds to the heavy chains of IgG after incubation with DTT; the L—band in the region of 25 kDa, which corresponds to the light chains of IgG after incubation with DTT.

In addition, we present the results of the determination of proteolytic activity *in situ.* To exclude possible hypothetical traces of contaminating canonical proteases, the IgG preparations were separated by SDS-PAGE, and their MBP-hydrolyzing activity was detected after extraction of the proteins from the separated gel slices only in the IgG band, while the other gel fragments were catalytically inactive (Figure 4). The electrophoretic mobility of usually low molecular mass canonical proteases (24–25 kDa) cannot coincide with that of intact IgGs (150 kDa).

Therefore, the detection of protease activity in the gel fragments corresponding only to intact IgGs (Figure 4) together with data of gel filtration of the Abs under acid-shock conditions (Figure 3) and with the absence of any other proteins and bands (Figures 1 and 2) provides direct evidence that the IgGs have MBP-hydrolyzing activity.

### 3.2. Аnalysis Of the Product Profile of MBP Hydrolysis by Antibodies of Patients with Schizophrenia

**Figure 5:**
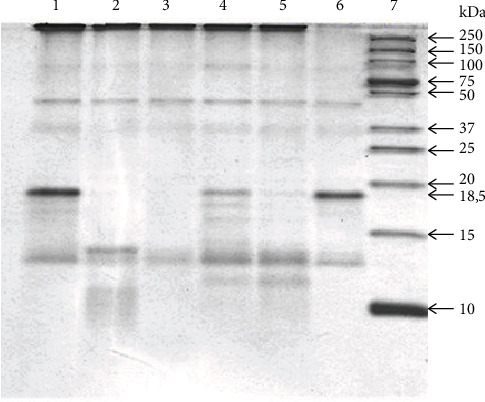
Electrophoretic analysis of MBP-hydrolyzing activity of IgG in SDS-PAGE 12.5% gel: 1-5 products profile of MBP-hydrolysis by individual IgG of patients with schizophrenia (line 1,4—low and medium rate of MBP-hydrolyzing activity; line 2,3,5—high rate of MBP-hydrolyzing activity); 6—control: MBP incubated without IgG; 7—protein molecular mass markers.

Various molecular products are formed during the MBP hydrolysis by the catalytic IgG of schizophrenia patients. The MBP hydrolysis was accompanied by the appearance of new bands of MBP products that are not represented in the control in the molecular weight regions corresponding to 16.0 kDa, 14.3 kDa, 12.7 kDa, 11.5 kDa, 10.7 kDa, 9.7 kDa, and 7.4 kDa (Figure 5). A change in the color intensity of the bands represented in control was detected: in the area of molecular weight 18.5 kDa—a decrease in color intensity and band size; in the area of molecular weight 13.5 kDa and 12.8 kDa—an increase in color intensity and band size.

The spectrum of MBP hydrolysis products depended on the level of IgG activity. High activity IgGs were characterized by the formation of numerous low molecular weight products that were located in the 13.5-7.0 kDa region. MBP-products profile after hydrolysis by antibodies with medium or low activity was characterized by products with a molecular weight of 18.5-12.0 kDa. Thus, highly active antibodies almost completely hydrolyzed the band of intact MBP with an isoform of 18.5 kDa (the main isoform of this protein for humans). While in electrophoretic tracks corresponding to antibodies with medium or low activity, the band 18.5 kDa remains clearly visible. For this reason, the activity level of catalytic IgG was assessed by the reduction of the color intensity of the bands in the region of 18.5 kDa.

Thus, the spectrum of products of the reaction of hydrolysis of MBP is extremely variable from person to person.

### 3.3. Proteolytic Activity of Autoantibodies to MBP in Sera of Schizophrenia Patients

We evaluated the proteolytic activities of polyclonal antibodies to MBP in the sera of schizophrenia patients. After incubation of MBP with IgG, the proteins were separated using gel electrophoresis and analyzed. The reaction conditions and the detection of the hydrolysis products are described in Section 2.

Analysis of our data showed that most patients had significant activity, while IgG from healthy controls had almost no such activity. Furthermore, the level of Abs activity was different depending on the type of the disease course.

Our results demonstrate that the specific anti-MBP IgGs from patients with schizophrenia catalyze the hydrolysis of MBP. The IgGs with the highest proteolytic activity are present in the analyzed serum samples of patients with acute schizophrenia, and the activity was twice higher than in patients in remission and significantly higher than in IgGs from healthy controls (Table 3). Thus, we conclude that the level of MBP hydrolysis by IgG of the schizophrenia patients decreased simultaneously with a decrease in the severity of their clinical symptoms. This was confirmed by their PANSS scores.

**Figure 6:**
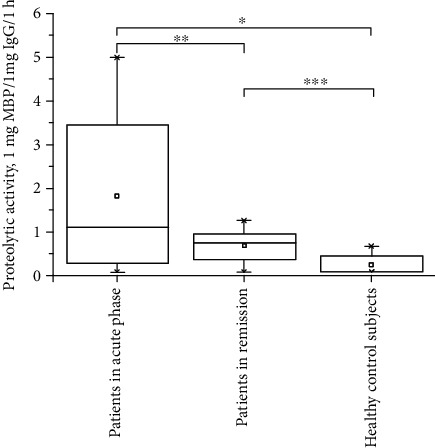
Levels of proteolytic activity in hydrolysis of MBP corresponding to IgG from sera of patients with acute schizophrenia, patients in remission, and healthy individuals; (—) median of catalytic activity; (between-group differences: ^∗^*p* = 0.000009; ^∗∗^*p* = 0.000127; ^∗∗∗^*p* = 0.000037 (by the Mann–Whitney *U* test).

Comparison of MBP-hydrolyzing activity in IgGs using the Mann–Whitney *U* test showed the greatest difference between the groups of healthy controls and patients in acute schizophrenia (*p* = 0.000009); between healthy individuals and schizophrenia patients in remission (*p* = 0.000037); and between patients in the acute phase and patients in remission (*p* = 0.000127) (Figure 6).

Comparison of the level of MBP-hydrolyzing activity of Abs in groups with paranoid schizophrenia (median [Q1; Q2] 1.042 [0.123; 4.156] mg MBP/mg IgG/h) and simple schizophrenia (0.630 [0.309; 2.05] mg MBP/mg IgG/h) revealed no significant differences (*p* = 0.334).

A more detailed analysis of the MBP-hydrolyzing activity of patients with paranoid schizophrenia revealed that the activity differs significantly depending on the type of disease course (Table 4).

All groups of paranoid schizophrenia with different courses and simple schizophrenia also showed significant differences from the control group (Table 5).

Thus, the highest activity was found in patients with the continuous course of schizophrenia (median: 1.810 mg MBP/mg IgG/h), which differed significantly from that in patients in remission (*p* = 0.000729; Mann–Whitney *U* test) and in controls (*р* = 0.000038; Mann–Whitney *U* test). This result also coincided with the maximum PANSS score in patients with the continuous course of schizophrenia.

Multiple lines of evidence now suggest damage to oligodendroglia and myelin in schizophrenia patients. This is confirmed by genetic, morphological, immunohistochemical, and neuroimaging studies. Expression levels of a number of myelin-related genes were significantly downregulated in schizophrenic brains including myelin-associated glycoprotein (MAG), CNP, myelin and lymphocyte protein (MAL), gelsolin (CSN), ErbB3 (also called HER3), and transferrin [39–46]. It is known that MBP reflects the myelin-forming activity of oligodendroglia through a positive correlation with the number of normal myelin fibers [16, 17]. Postmortem studies show a decrease in the expression of MBP mRNA and protein levels in various regions of the brain [10, 11, 14]. Morphological studies also demonstrate ultrastructural anomalies of myelin fibers in schizophrenia patients according to studies on postmortem autopsy samples of the brain [12, 13]. These changes were most pronounced in the prefrontal cortex and reliably correlated with the degree of expression in the caudate nucleus and hippocampus. A decrease in the density of white matter oligodendrocytes in the postmortem frontal cortex was described for a small sample group of elderly schizophrenia patients, but myelin protein expression was not quantified for white matter [47]. Data of neuroimaging studies suggest that white matter abnormalities are present even before the onset of the illness but may be a stable characteristic of the disease. Changes in brain structure might be formed immediately after the first episode of schizophrenia and increase with the course of the disease [48–52].

Antibodies to MBP and an increase in the MBP level were detected in cerebrospinal fluid (CSF) and serum of patients with schizophrenia [53]. However, the total level of immunoglobulin G in both CSF and peripheral blood of patients with schizophrenia was not always found to be different from that in healthy individuals, and sometimes it was found to be lower than in healthy individuals [54, 55]. In studies with experimental animals, it was noted that autoantibodies binding neuroantigens in the early stages of ontogenesis contribute to the formation of various structural anomalies in the nervous system and subsequently lead to a violation of the behavior of young animals. Thus, a high level of antibodies to nerve growth factor in the blood of pregnant females has a pronounced inhibitory effect on the learning process and significantly increases the threshold of pain sensitivity in the offspring [8, 56, 57]. Considering the data on the penetration of immunoglobulins through the blood-brain barrier in autoimmune disorders [58, 59] and the detected BBB hyperpermeability in schizophrenia [60], this phenomenon can be explained by the fact that MBP stimulates the synthesis of antibodies to myelin components. Also, it is generally accepted that the detection of MBP in blood is indicative of impairment of myelination in the brain, but the reason for this is not established.

Our results demonstrate that IgGs from schizophrenia patients catalyze the hydrolysis of MBP, and this activity is significantly higher than in control subjects. We have found that the activity differs significantly depending on the type of the disease course, particularly patients with paranoid schizophrenia and continuous course of schizophrenia demonstrated the highest activity (1.810 mg MBP/mg IgG/h), which was significantly different from that in patients in remission and the control. Signs of continuous course are expressed in the regular development, complication, and deterioration of symptoms as the disease progresses, which affects the immunological reactivity of the organism on the whole. Our data suggest that in patients with a continuous course of schizophrenia, the violation of myelination is perhaps more pronounced.

According to the results of our colleagues from the Laboratory of Clinical Psychoneuroimmunology, specific features of dysregulation of the immune system in patients with schizophrenia were identified. Suppression of the T-cellular immune response (decrease CD2+, CD3+, CD4+, CD16+ subtypes of T-lymphocytes) and activation of humoral immunity (increase in levels of circulating immune complexes, IgM levels, and B-lymphocytes), a disturbance of cytokine production was revealed [61]. It should be noted that patients with a continuous course of schizophrenia have more pronounced changes in their immune system. In particular, they have simultaneous activation of Th1 and Th2 cytokines with the dominance profile of the Th1-cell-response pathway [62]. Thus, we assume that the proteolytic activity of Abs in MBP hydrolysis demonstrates the course of autoimmune reactions in patients with a continuous course of schizophrenia. According to recent studies, the immunological basis of schizophrenia is like that of chronic inflammation [63–66]. Proteolytic IgG in patients with schizophrenia can also play a positive role by hydrolyzing MBP as a potential immunogen in peripheral blood, since in some other diseases, catalytic antibodies can perform a protective function by hydrolyzing pathological proteins [67–70].

Since we cannot completely exclude the effect of antipsychotic drugs in our study, we must consider their potential impact on the immune response and catalytically activity. Clinical trials of risperidone have shown that the drug can reduce circulating levels of IL-1*β* in patients with schizophrenia, as well as increase the levels of neurotrophin BDNF [71]. Risperidone, clozapine, and haloperidol have a positive correlation with improved clinical symptoms of schizophrenia and decreased levels of circulating IL-2, IL-6, IL-18, IFN-*γ*, and TNF-*α* [72–74]. Haloperidol treatment inhibits the immune response by suppressing NF-*κ*B signaling via the dopamine D2 receptor and inhibition of proinflammatory cytokine release [75, 76]. Moreover, according to the results of our study, proteolytic activity was maximal before the prescription of antipsychotic therapy and decreased after drug treatment in the remission group. Thereby, we suggest it is unlikely that the induction of catalytic activity is caused by the antipsychotics.

## 4. Conclusions

Our results demonstrate that IgGs from schizophrenia patients catalyze the hydrolysis of MBP, and this activity is significantly higher than IgGs from control subjects. We found that the activity level differs depending on the type of the disease course, particularly in patients with paranoid schizophrenia with continuous course demonstrating the highest activity level (1.810 mg MBP/mg IgG/h), which significantly exceeded the activity level in patients in remission and control. Thus, we suggest that patients with a continuous type of schizophrenia have a more pronounced violation of the myelination in brain structures. This reflects the peculiarity of the immunological reactivity of the organism as a whole of the continuous type of schizophrenia.

## Figures and Tables

**Table 1:** Demographic and clinical characteristics of schizophrenia patients and healthy control subjects.

	Healthy control subjects	Patients with acute schizophrenia	Patients with schizophrenia in remission	*p* value Mann–Whitney *U* test
Number	24	48	31	NS
Age median (IQR)	39.00 [29.00; 46.00]	38.00 [30.00; 46.00]	35.00 [31.00; 44.00]	NS
Male/female	9/15	12/28	16/15	NS
Duration of disease, years median (IQR)		12.50 [6.00; 17.00]	15.00 [10.00; 18.00]	NS
PANSS total score		89.00 [80.00; 105.00]	54.50 [52.00; 59.00]	0.0001

^∗^NS: nonsignificant between the group of healthy control subjects and different groups of patients *р* = 0.0001 between the groups of patients with acute schizophrenia and patients in remission.

**Table 2:** Demographic and clinical characteristics of patients with different types of schizophrenia.

Subgroups of patients with schizophrenia	Continuous course of schizophrenia	Episodic course of schizophrenia with progressive deficit	Episodic course of schizophrenia with stable deficit	Simple schizophrenia	*p* value Kruskal–Wallis test
Number	12	12	12	12	NS
Age median (IQR)	34.00 [32.00; 54.00]	39.00 [38.00; 46.00]	39.00 [34.00; 41.00]	34.00 [26.00; 45.00]	NS
Male/female	4/7	3/8	2/7	3/6	NS
Duration of disease, years median (IQR)	14.00 [13.00; 20.00]	13.00 [6.00; 17.00]	11.00 [5.00; 12.00]	8.00 [5.00; 17.00]	NS
PANSS total score	100.00 [87.00; 116.00]	83.00 [74.00; 98.00]	76.00 [65.00; 81.00]	92.00 [84.00; 111.00]	0.0381

^∗^NS: nonsignificant; *p* value for multiple comparisons with Kruskal–Wallis test including all groups of patients.

**Table 3:** MBP-hydrolyzing activity levels in IgGs from schizophrenia patients and healthy control subjects.

Healthy control subjects, median [Q1-Q4] mg MBP/mg IgG/h	Patients with acute schizophrenia, median [Q1-Q4] mg MBP/mg IgG/h	Patients with schizophrenia in remission, median [Q1-Q4] mg MBP/mg IgG/h	*p* value Kruskal–Wallis test
*N* = 24	*N* = 48	*N* = 31	
0.00 [0.00; 0.24]	1.026 [0.205; 3.372]	0.656 [0.279; 0.873]	0.0001^∗^

^∗^ *p* value from multiple comparisons with Kruskal–Wallis test including all groups of patients and healthy control group; *p* significant at <0.05.

**Table 4:** Characteristics of IgG proteolytic activity levels in patients with different clinical forms of schizophrenia and in healthy control subjects.

Group	Healthy control subjects, mg MBP/mg IgG/h	Patients with remission, mg MBP/mg IgG/h	Continuous course of schizophrenia, mg MBP/mg IgG/h	Episodic course of schizophrenia with progressive deficit, mg MBP/mg IgG/h	Episodic course of schizophrenia with stable deficit, mg MBP/mg IgG/h	Simple schizophrenia, mg MBP/mg IgG/h	*p* value
*N*	24	31	12	12	12	12	
Median [Q1-Q4]	0.00[0.00; 0.24]	0.656[0.279; 0.873]	1.810[0.746; 4.101]	0.539[0.00; 4.460]	0.831[0.123; 1.859]	0.630[0309; 2.050]	0.0002^∗^

^∗^ *p* value from multiple comparisons with Kruskal–Wallis test including all groups of patients and healthy control group; *p* significant at <0.05.

**Table 5:** Significance of differences in IgG proteolytic activity in patients with acute schizophrenia and patients with schizophrenia in remission and healthy control subjects, *p* value calculated using the Mann–Whitney *U* test.

Code for group of patients	Episodic course of schizophrenia with progressive deficit	Episodic course of schizophrenia with stable deficit	Simple schizophrenia	Patients with remission	Healthy control subjects
Continuous course of schizophrenia	0.187706	0.223449	0.183506	0.000729^∗^	0.000038^∗^
Episodic course of schizophrenia with progressive deficit		0.592055	0.731468	1.000000	0.011796^∗^
Episodic course of schizophrenia with stable deficit			1.000000	0.398951	0.004769^∗^
Simple schizophrenia				0.475707	0.001954^∗^

^∗^ *p* value from pairwise comparison with Mann–Whitney *U* test between groups, which are named in the corresponding row and column headings for each cell; *p* < 0.05.

## Data Availability

Data used to support the findings of this study are available from the corresponding author upon request.

## References

[1] Strous R. D., Shoenfeld Y. (2006). Schizophrenia, autoimmunity and immune system dysregulation: a comprehensive model updated and revisited. *Journal of Autoimmunity*.

[2] Eaton W. W., Byrne M., Ewald H. (2006). Association of schizophrenia and autoimmune diseases: linkage of Danish national registers. *American Journal of Psychiatry*.

[3] Tiosano S., Farhi A., Watad A. (2017). Schizophrenia among patients with systemic lupus erythematosus: population-based cross-sectional study. *Epidemiology and Psychiatric Sciences*.

[4] Mack A., Pfeiffer C., Schneider E. M., Bechter K. (2017). Schizophrenia or atypical lupus erythematosus with predominant psychiatric manifestations over 25 years: case analysis and review. *Frontiers in Psychiatry*.

[5] Drexhage R. C., Hoogenboezem T. A., Cohen D. (2011). An activated set point of T-cell and monocyte inflammatory networks in recent-onset schizophrenia patients involves both pro- and anti-inflammatory forces. *International Journal of Neuropsychopharmacology*.

[6] Otman I. N., Zozulya S. A., Sarmanova Z. V., Klushnik T. P. (2015). Inflammatory and autoimmune reactions in different forms of nervous system functioning disorders. *Patologicheskaia fiziologiia i eksperimental'naia terapiia*.

[7] Muller N., Schwarz M. J. (2010). Immune system and schizophrenia. *Current Immunology Reviews*.

[8] Morozov S. G., Gribova I. E., Klushnik T. P. (2007). Influence of high level of antibodies to myelin basic protein in female mice on the postnatal development and behavioral reactions of the progeny. *Bulletin of Experimental Biology and Medicine*.

[9] Raabe F. J., Galinski S., Papiol S., Falkai P. G., Schmitt A., Rossner M. J. (2018). Studying and modulating schizophrenia-associated dysfunctions of oligodendrocytes with patient-specific cell systems. *NPJ Schizophrenia*.

[10] Martins-de-Souza D., Gattaz W. F., Schmitt A. (2009). Proteomic analysis of dorsolateral prefrontal cortex indicates the involvement of cytoskeleton, oligodendrocyte, energy metabolism and new potential markers in schizophrenia. *Journal of Psychiatric Research*.

[11] Martins-de-Souza D. (2010). Proteome and transcriptome analysis suggests oligodendrocyte dysfunction in schizophrenia. *Journal of Psychiatric Research*.

[12] Kolomeets N. S., Uranova N. A. (2008). Pathology of oligodendroglia and myelinated fibers of the hippocampus in schizophrenia (an ultrastructural and morphometric study). *Zhurnal Nevrologii i Psikhiatrii Imeni S.S. Korsakova*.

[13] Uranova N. A., Vostrikov V. M., Orlovskaya D. D., Rachmanova V. I. (2004). Oligodendroglial density in the prefrontal cortex in schizophrenia and mood disorders: a study from the Stanley Neuropathology Consortium. *Schizophrenia Research*.

[14] Matthews P. R., Eastwood S. L., Harrison P. J. (2012). Reduced myelin basic protein and actin-related gene expression in visual cortex in schizophrenia. *PLoS ONE*.

[15] Honer W. G., Falkai P., Chen C., Arango V., Mann J. J., Dwork A. J. (1999). Synaptic and plasticity-associated proteins in anterior frontal cortex in severe mental illness. *Neuroscience*.

[16] Burbaeva G. S., Boksha I. S., Tereshkina E. B. (2007). Systemic neurochemical alterations in schizophrenic brain: glutamate metabolism in focus. *Neurochemical Research*.

[17] Boksha I. (2012). *Specific Metabolism of Glutamate in Schizophrenia*.

[18] Rimon R., Ahokas A., Ruutiainen J., Halonen P. (1986). Myelin basic protein antibodies in catatonic schizophrenia. *The Journal of Clinical Psychiatry*.

[19] Klyushnik T. P., Siriachenko T. M., Sarmanova Z. V., Otman I. N., Dupin A. M., Sokolov R. E. (2008). Changes of the level of serum antibodies to neuroantigens in patients with schizophrenia during the treatment. *Zhurnal Nevrologii i Psikhiatrii Imeni S.S. Korsakova*.

[20] Goldsmith C. A., Rogers D. P. (2008). The case for autoimmunity in the etiology of schizophrenia. *Pharmacotherapy*.

[21] Khandaker G. M., Cousins L., Deakin J., Lennox B. R., Yolken R., Jones P. B. (2015). Inflammation and immunity in schizophrenia: implications for pathophysiology and treatment. *The Lancet Psychiatry*.

[22] van Rees G. F., Lago S. G., Cox D. A. (2018). Evidence of microglial activation following exposure to serum from first-onset drug-naïve schizophrenia patients. *Brain, Behavior, and Immunity*.

[23] Bergink V., Gibney S. M., Drexhage H. A. (2014). Autoimmunity, inflammation, and psychosis: a search for peripheral markers. *Biological Psychiatry*.

[24] Polosukhina D. I., Kanyshkova T. G., Doronin B. M. (2004). Hydrolysis of myelin basic protein by polyclonal catalytic IgGs from the sera of patients with multiple sclerosis. *Journal of Cellular and Molecular Medicine*.

[25] Bezuglova A. M., Konenkova L. P., Doronin B. M., Buneva V. N., Nevinsky G. A. (2011). Affinity and catalytic heterogeneity and metal-dependence of polyclonal myelin basic protein-hydrolyzing IgGs from sera of patients with systemic lupus erythematosus. *Journal of Molecular Recognition*.

[26] Gonzalez-Gronow M., Cuchacovich M., Francos R. (2015). Catalytic autoantibodies against myelin basic protein (MBP) isolated from serum of autistic children impair *in vitro* models of synaptic plasticity in rat hippocampus. *Journal of Neuroimmunology*.

[27] Parshukova D., Smirnova L. P., Ermakov E. A. (2019). Autoimmunity and immune system dysregulation in schizophrenia: IgGs from sera of patients hydrolyze myelin basic protein. *Journal of Molecular Recognition*.

[28] Grodzki A. C., Berenstein E. (2010). Antibody purification: affinity chromatography–protein A and protein G Sepharose. *Immunocytochemical methods and protocols*.

[29] Ermakov E. A., Smirnova L. P., Parkhomenko T. A. (2015). DNA-hydrolysing activity of IgG antibodies from the sera of patients with schizophrenia. *Open Biology*.

[30] Ermakov E. A., Smirnova L. P., Bokhan N. A. (2017). Catalase activity of IgG antibodies from the sera of healthy donors and patients with schizophrenia. *PLoS ONE*.

[31] Laemmli U. K. (1970). Cleavage of structural proteins during the assembly of the head of bacteriophage T4. *Nature*.

[32] Baranovskii A. G., Ershova N. A., Buneva V. N. (2001). Catalytic heterogeneity of polyclonal DNA-hydrolyzing antibodies from the sera of patients with multiple sclerosis. *Immunology Letters*.

[33] Ponomarenko N. A., Durova O. M., Vorobiev I. I. (2006). Autoantibodies to myelin basic protein catalyze site-specific degradation of their antigen. *Proceedings of the National Academy of Sciences*.

[34] Sapparapu G., Planque S. A., Nishiyama Y., Foung S. K., Paul S. (2009). Antigen-specific proteolysis by hybrid antibodies containing promiscuous proteolytic light chains paired with an antigen-binding heavy chain. *Journal of Biological Chemistry*.

[35] Wootla B., Christophe O. D., Mahendra A. (2011). Proteolytic antibodies activate factor IX in patients with acquired hemophilia. *Blood*.

[36] Tolmacheva A. S., Blinova E. A., Ermakov E. A., Buneva V. N., Vasilenko N. L., Nevinsky G. A. (2015). IgG abzymes with peroxidase and oxidoreductase activities from the sera of healthy humans. *Journal of Molecular Recognition*.

[37] Schauenstein E. S., Dachs F., Reiter M., Gombotz H., List W. (1986). Labile Disulfide Bonds and Free Thiol Groups in Human IgG I. *International Archives of Allergy and Immunology*.

[38] Schurman J., Perdok G. J., Gorter A. D., Aalberse R. C. (2001). The inter-heavy chain disulfide bonds of IgG4 are in equilibrium with intra-chain disulfide bonds. *Molecular Immunology*.

[39] Davis K. L., Haroutunian V. (2003). Global expression-profiling studies and oligodendrocyte dysfunction in schizophrenia and bipolar disorder. *The Lancet*.

[40] Takahashi N., Sakurai T., Davis K. L., Buxbaum J. D. (2011). Linking oligodendrocyte and myelin dysfunction to neurocircuitry abnormalities in schizophrenia. *Progress in Neurobiology*.

[41] Hakak Y., Walker J. R., Li C. (2001). Genome-wide expression analysis reveals dysregulation of myelination-related genes in chronic schizophrenia. *Proceedings of the National Academy of Sciences*.

[42] Aston C., Jiang L., Sokolov B. P. (2004). Microarray analysis of postmortem temporal cortex from patients with schizophrenia. *Journal of Neuroscience Research*.

[43] Aston C., Jiang L., Sokolov B. P. (2005). Transcriptional profiling reveals evidence for signaling and oligodendroglial abnormalities in the temporal cortex from patients with major depressive disorder. *Molecular Psychiatry*.

[44] Dracheva S., Davis K. L., Chin B., Woo D. A., Schmeidler J., Haroutunian V. (2006). Myelin-associated mRNA and protein expression deficits in the anterior cingulate cortex and hippocampus in elderly schizophrenia patients. *Neurobiology of Disease*.

[45] Katsel P., Davis K. L., Haroutunian V. (2005). Variations in myelin and oligodendrocyte-related gene expression across multiple brain regions in schizophrenia: a gene ontology study. *Schizophrenia Research*.

[46] Tkachev D., Mimmack M. L., Ryan M. M. (2003). Oligodendrocyte dysfunction in schizophrenia and bipolar disorder. *The Lancet*.

[47] Beasley C. L., Dwork A. J., Rosoklija G. (2009). Metabolic abnormalities in fronto-striatal-thalamic white matter tracts in schizophrenia. *Schizophrenia Research*.

[48] Cahn W., Pol H. E. H., Lems E. B. (2002). Brain Volume Changes in First-Episode Schizophrenia:A 1-Year Follow-up Study. *Archives of General Psychiatry*.

[49] Deng M. Y., McAlonan G. M., Cheung C. (2009). A naturalistic study of grey matter volume increase after early treatment in anti-psychotic naïve, newly diagnosed schizophrenia. *Psychopharmacology*.

[50] Moreno D., Burdalo M., Reig S. (2005). Structural neuroimaging in adolescents with a first psychotic episode. *Journal of the American Academy of Child & Adolescent Psychiatry*.

[51] Sun D., Stuart G. W., Jenkinson M. (2009). Brain surface contraction mapped in first-episode schizophrenia: a longitudinal magnetic resonance imaging study. *Molecular Psychiatry*.

[52] Witthaus H., Mendes U., Brüne M. (2010). Hippocampal subdivision and amygdalar volumes in patients in an at-risk mental state for schizophrenia. *Journal of Psychiatry and Neuroscience*.

[53] Li S., Wu H., Guo H., Zhao Z. (2006). Neuron-specific Enolase and myelin basic protein in cerebrospinal fluid of patients with first episode schizophrenia. *Journal of Huazhong University of Science and Technology. Medical Sciences*.

[54] Melkersson K., Bensing S. (2018). Signs of impaired blood-brain barrier function and lower IgG synthesis within the central nervous system in patients with schizophrenia or related psychosis, compared to that in controls. *Neuro Endocrinology Letters*.

[55] Glass L. J., Sinclair D., Boerrigter D. (2017). Brain antibodies in the cortex and blood of people with schizophrenia and controls. *Translational Psychiatry*.

[56] Zuckerman L., Weiner I. (2005). Maternal immune activation leads to behavioral and pharmacological changes in the adult offspring. *Journal of Psychiatric Research*.

[57] Klyushnik T. P., Krasnolobova S. A., Sarmanova Z. V., Shcherbakova I. V., Morozov S. G., Gribova I. E. (2004). Effect of antibodies to nerve growth factor and serum albumin on the development and behavior of mice. *Bulletin of Experimental Biology and Medicine*.

[58] Brimberg L., Mader S., Fujieda Y. (2015). Antibodies as mediators of brain pathology. *Trends in Immunology*.

[59] St-Amour I., Paré I., Alata W. (2013). Brain bioavailability of human intravenous immunoglobulin and its transport through the murine blood–brain barrier. *Journal of Cerebral Blood Flow & Metabolism*.

[60] Najjar S., Pahlajani S., De Sanctis V., Stern J. N., Najjar A., Chong D. (2017). Neurovascular unit dysfunction and blood–brain barrier hyperpermeability contribute to schizophrenia neurobiology: a theoretical integration of clinical and experimental evidence. *Frontiers in Psychiatry*.

[61] Vetlugina T. P., Lobacheva O. A., Semke A. V., Nikitina V. B., Bokhan N. A. (2016). An effect of quetiapine on the immune system of patients with schizophrenia. *Zhurnal nevrologii i psikhiatrii im. S.S. Korsakova*.

[62] Abrosimova Y. S. (2007). Cytokine disbalance and Fas-dependent apoptosis in shift-like and continued schizophrenia. *Neurological bulletin named after Vladimir Bekhterev*.

[63] Torrey E. F., Yolken R. H. (2001). The schizophrenia–rheumatoid arthritis connection: infectious, immune, or both?. *Brain, Behavior, and Immunity*.

[64] Hardoy M. C., Cadeddu M., Serra A. (2011). A pattern of cerebral perfusion anomalies between major depressive disorder and Hashimoto thyroiditis. *BMC Psychiatry*.

[65] Müller N. (2018). Inflammation in schizophrenia: pathogenetic aspects and therapeutic considerations. *Schizophrenia Bulletin*.

[66] Bauer M. E., Teixeira A. L. (2019). Inflammation in psychiatric disorders: what comes first?. *Annals of the New York Academy of Sciences*.

[67] Taguchi H., Planque S., Nishiyama Y. (2008). Autoantibody-catalyzed hydrolysis of amyloid *β* peptide. *Journal of Biological Chemistry*.

[68] Odintsova E. S., Baranova S. V., Dmitrenok P. S. (2011). Antibodies to HIV integrase catalyze site-specific degradation of their antigen. *International Immunology*.

[69] Nevinsky G. A. (2011). Natural catalytic antibodies in norm and in HIV-infected patients. *Understanding HIV/AIDS management and care—pandemic approaches the 21st century*.

[70] Nevinsky G. A., Buneva V. N. (2010). Natural catalytic antibodies in norm, autoimmune, viral, and bacterial diseases. *The Scientific World Journal*.

[71] Chen S.-J., Chao Y.-L., Chen C.-Y. (2012). Prevalence of autoimmune diseases in in-patients with schizophrenia: nationwide population-based study. *The British Journal of Psychiatry*.

[72] Cazzullo C. L., Sacchetti E., Galluzzo A. (2002). Cytokine profiles in schizophrenic patients treated with risperidone: a 3-month follow-up study. *Progress in Neuro-Psychopharmacology and Biological Psychiatry*.

[73] Zhang X. Y., Zhou D. F., Cao L. Y., Zhang P. Y., Wu G. Y., Shen Y. C. (2004). Changes in serum interleukin-2, -6, and -8 levels before and during treatment with risperidone and haloperidol. *The Journal of Clinical Psychiatry*.

[74] Zhang X. Y., Zhou D. F., Cao L. Y., Wu G. Y., Shen Y. C. (2005). Cortisol and cytokines in chronic and treatment-resistant patients with schizophrenia: association with psychopathology and response to antipsychotics. *Neuropsychopharmacology*.

[75] McKenna F., McLaughlin P. J., Lewis B. J. (2002). Dopamine receptor expression on human T-and B-lymphocytes, monocytes, neutrophils, eosinophils and NK cells: a flow cytometric study. *Journal of Neuroimmunology*.

[76] Yamamoto S., Ohta N., Matsumoto A., Horiguchi Y., Koide M., Fujino Y. (2016). Haloperidol suppresses NF-kappaB to inhibit lipopolysaccharide-induced pro-inflammatory response in RAW 264 cells. *Medical Science Monitor*.

